# Current Understanding of Circular RNAs in Systemic Lupus Erythematosus

**DOI:** 10.3389/fimmu.2021.628872

**Published:** 2021-02-25

**Authors:** Hongjiang Liu, Yundong Zou, Chen Chen, Yundi Tang, Jianping Guo

**Affiliations:** ^1^Department of Rheumatology and Immunology, Peking University People’s Hospital & Beijing Key Laboratory for Rheumatism Mechanism and Immune Diagnosis (BZ0135), Beijing, China; ^2^Department of Rheumatology and Immunology, The People’s Hospital of China Three Gorges University/The First People’s Hospital of Yichang, Yichang, China

**Keywords:** circRNAs, endogenous regulation, biological function, biomarker, systemic lupus erythematosus

## Abstract

Systemic lupus erythematosus (SLE) is a common and potentially fatal autoimmune disease that affects multiple organs. To date, its etiology and pathogenesis remains elusive. Circular RNAs (circRNAs) are a novel class of endogenous non-coding RNAs with covalently closed loop structure. Growing evidence has demonstrated that circRNAs may play an essential role in regulation of gene expression and transcription by acting as microRNA (miRNA) sponges, impacting cell survival and proliferation by interacting with RNA binding proteins (RBPs), and strengthening mRNA stability by forming RNA-protein complexes duplex structures. The expression patterns of circRNAs exhibit tissue-specific and pathogenesis-related manner. CircRNAs have implicated in the development of multiple autoimmune diseases, including SLE. In this review, we summarize the characteristics, biogenesis, and potential functions of circRNAs, its impact on immune responses and highlight current understanding of circRNAs in the pathogenesis of SLE.

## Introduction

Systemic lupus erythematosus (SLE) is a chronic systemic autoimmune disease characterized by production of multiple autoantibodies and involvement of multi-systemic organ damage (1). Although genetic and environmental factors have been implicated in the development of SLE, its exact etiology and pathogenesis remain unclear (2). Furthermore, the early diagnosis and effective therapies for SLE have been a challenge, which maybe partly attributed to the poor understanding of the disease pathogenesis. Therefore, dissecting the molecular mechanisms underlying SLE pathogenesis will provide clues for identifying novel diagnostic markers and therapeutics for SLE patients. Recent evidence indicates that the non-coding RNAs (ncRNAs), such as the microRNAs (miRNAs), long non-coding RNAs (lncRNAs), and circular RNAs (circRNAs), can function as epigenetic factors and play important roles in the development and progression of SLE (3, 4). Among these non-coding RNAs, the circRNAs are the novel class of ncRNAs with covalently closed RNA circles by non-canonical backsplicing events (5). These circular RNAs are highly conserved, stable, and resistant to RNase (6, 7). Thus, the circRNAs could be considered as better biomarkers than the linear ncRNAs for diagnosis and management of diseases. In this review, we summarize circRNA biogenesis, classification, biological functions and its implication in immune responses and diseases, with a particular emphasis on the emerging roles of circRNAs in SLE.

## CircRNA Discovery

CircRNAs were first discovered in pathogens in 1976 (8). In the study, Sanger and colleagues characterized the structure for viroids, which are single-stranded and covalently closed circular RNA molecules. A few years later, a second study observed that circRNAs were existed in the cytoplasm of eukaryotic cell lines with an electron microscopy (9). Thereafter, a handful of circRNAs were reported in the early 1990 (10–12). However, these findings were not followed up, as at the time circRNAs were mainly considered to be byproducts generated by splicing error of pre-mRNA processing without significant biological effect (13). Until recent years, with the advance of high-throughput sequencing and bioinformatics algorithms, researchers have identified thousands of circRNAs in eukaryotes and found some circRNAs expressed in a tissue-specific manner with potential functions (14–16).

Since the landmark finding of CDR1as and circSry in 2013, which could function as microRNA sponge (17, 18), the circRNAs have become a novel hotspot in non-coding RNAs (19). Accumulating evidence has demonstrated that circRNAs are an abundant and conserved subclass of RNAs and are widely expressed in a cell-/tissue-specific, and development-dependent manner (15, 16, 20–22). As stated before, the circRNAs are distinguished from the linear RNAs by the structure of covalently closed loop lacking 5’ cap and 3’ poly-adenylated tails. This unique feature makes the circRNAs much more stable than their linear counterparts and resistant to degradation by exonuclease and RNases (6). Therefore, the circRNAs are proposed as novel and promising diagnostic biomarkers and potential therapeutic targets for diseases. So far, circRNAs have been reported to be involved in the pathogenesis of multiple diseases, such as neurological disorders, cardiovascular diseases, cancer, and chronic inflammatory and autoimmune diseases (23–26).

## CircRNA Biogenesis and Classification

**Figure 1:**
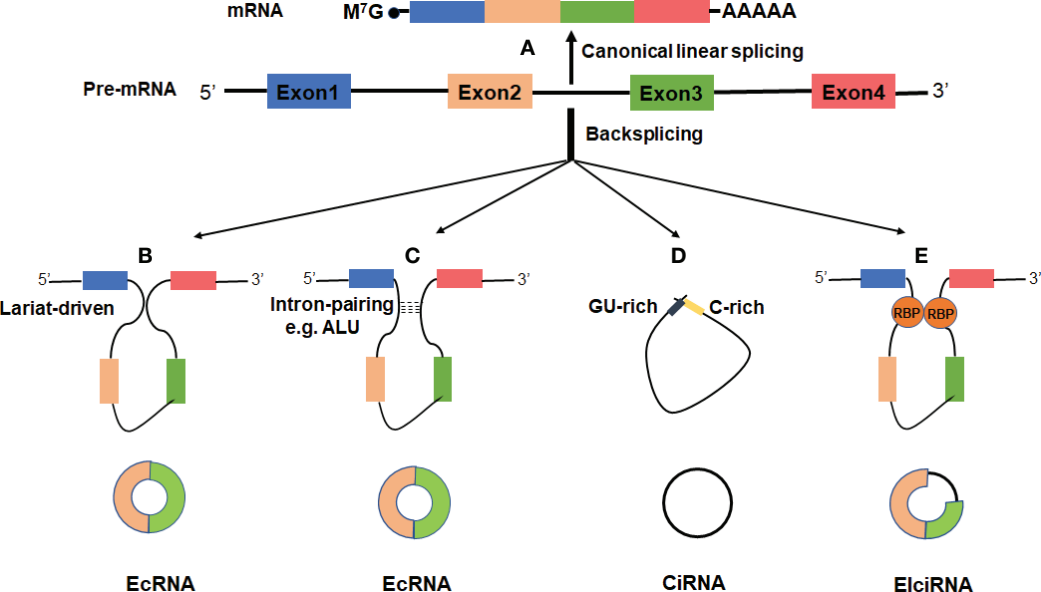
Biogenesis of three major types of circRNAs. **(A)** A linear mRNA is formed by canonical splicing from pre-messenger RNA (pre-mRNA). **(B–E)** CircRNAs are generated by backsplicing from pre-mRNA. **(B)** Lariat-driven circularization, exon-skipping leads to an exon-containing lariat whose restricted structure facilitates circularization. **(C)** Intro-pairing-driven circularization is mediated by base-pairing between inverted repeat elements such as ALU complementarity. **(D)** CiRNA biogenesis is mainly dependent on GU-rich motif and C-rich sequence. **(E)** RNA-binding proteins (RBP) serving as trans-acting factors can bring two splicing sites close together to promote circularization.

Alternative splicing of RNA is a key event for gene expression in eukaryotes. Precursor mRNA (pre-mRNAs) is processed by the spliceosome to remove introns and thereby exons are rapidly linked to form a functional mature mRNA (**Figure 1A**). It was widely accepted that circRNAs are derived from canonical splice sites (15, 18, 27). When pre-mRNA catalyzing events are slowed down, the nascent RNAs may be directed to alternative paths that accelerate back-splicing (5, 28). CircRNAs are generated by back-splicing that joins a 5’ splice donor to an upstream 3’ splice acceptor site, thus forming a single-strand covalently closed loop (15, 18, 29, 30). However, the exact mechanisms by which the spliceosome selects certain exons to circularize are not completely elucidated. Jeck and colleagues proposed two models for exon-derived circRNA formation: “lariat-driven circularization” and “intron-pairing-driven circularization” (15, 29). In the lariat-driven circulating model (**Figure 1B**), exon-skipping is required and leads to an exon-containing lariat, which will then itself be internally processed to form exon-derived circRNAs (15, 29). In intro-pairing-driven circularization (**Figure 1C**), cyclization is prompted presumably by intronic motifs such ALU complementarity bordering the circularized exon(s), which may bring the two exons (or the ends of the same exon) into close proximity to form the loop (15, 29). Meanwhile, Zhang et al. reported a novel subclass of circRNAs originating from introns (31). Such intronic circRNA generated from the intronic lariat contains a 7-nucleotide GU-rich element at the 5’ splice site and an 11-nucleotide C-rich element near the branchpoint site, which makes them resistant to the debranching enzyme and leads to the formation of an intro-derived circRNA (31) (**Figure 1D**). In some cases, if the intron between exons is retained, the cyclizing transcript tends to form exon-intron circRNAs (31, 32). In addition, some RNA binding proteins (RBPs) such as Quaking 1 (QKI) (33), muscleblind (MBL) (34), adenosine deaminase acting on RNA (ADAR) (35) and FUS (36) were reported to regulate the circRNA biogenesis (**Figure 1E**).

CircRNAs can be categorized into three major types based on their structural sequence: exonic circRNAs (EcRNAs), exon-intro circRNAs (ElciRNAs), and circular intronic RNAs (ciRNAs) (18, 31, 32). It has been observed that EcRNAs are predominantly localized in the cytoplasm, while ElciRNAs and ciRNAs are mainly restricted in the nucleus (18, 31, 32). EcRNAs are thought to be the most abundant circRNAs, which account for ~80% among currently discovered circRNAs (5, 37). In addition to the aforementioned types, it was reported that circRNAs can also be generated from intergenic genomic DNA (known as intergenic circRNAs) (18), the splicing of pre-tRNA [termed tRNA intronic circRNAs (tricRNAs)] (38), and from long ncRNAs (for example, circPINTexon2) (39).

## CircRNA Functions

**Figure 2:**
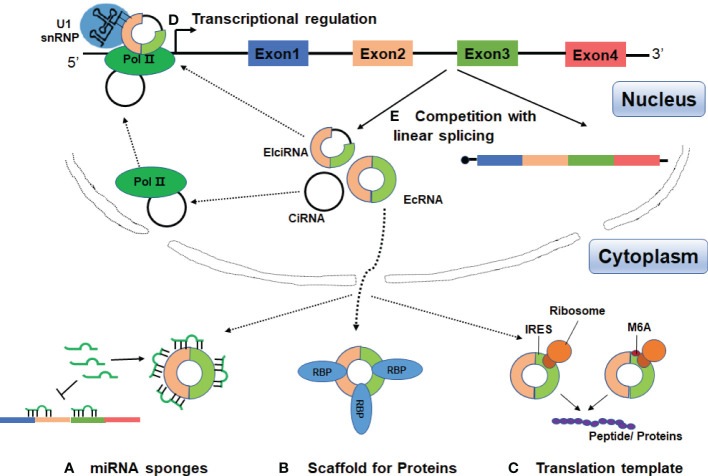
Functions of circRNAs. **(A)** CircRNAs can act as miRNA sponges to diminish the effects of miRNAs on target mRNAs. **(B)** CircRNAs can serve as scaffolds or decoys for RBPs to modulate their functions, thus affecting the related biological processes. **(C)** CircRNAs may be translated if they contain an internal ribosome entry site (IRES) or extensive N6-methyladenosines (m6A) modification. **(D)** CircRNAs can promote transcription by interacting with U1 small nuclear ribonucleoprotein (U1 snRNP) and the RNA polymerase II (Pol II) in the nucleus. **(E)** The processing of circRNAs could affect host gene expression by competing with their linear mRNA splicing.

In recent years, growing evidence indicates that circRNAs may play important roles in regulation of gene expression and participate in a series of pathophysiological processes (**Figure 2**).

### CircRNAs Act as Competing Endogenous RNA or miRNA Sponges

MicroRNAs (miRNAs) are a class of endogenous, noncoding RNAs of approximately 22 nucleotides in length, and can play crucial regulatory roles in post-transcription by targeting mRNAs for cleavage or translational repression (40). It has been proposed that the competing endogenous RNAs (ceRNAs) including pseudogenes and long-chain non-coding RNA (lncRNA) can act as sponges for miRNAs through their binding sites of miRNA recognition elements (MREs) (41–43). Subsequently, Hansen et al. reported that several abundant circRNAs can function as ceRNAs (17) (**Figure 2A**). CiRS-7 (also termed CDR1as) was first demonstrated to harbor more than 70 conserved binding sites for miR-7 (17, 18). Overexpression of ciRS7 can efficiently recruit miR-7 to promote the specific AGO2 interaction, thus resulting in decreased miR-7 function and up-regulation of its target mRNAs. Moreover, ciRS7 was highly expressed in brains and its function was conserved, overexpression of ciRS7 or knockdown of miR-7 could impair the development of midbrain and nerve in mice and zebrafish (17, 18). In esophageal squamous cell carcinoma, ciRS-7 accelerated tumor growth and metastasis by regulation of miR-7/HOXB13 (44).

Besides ciRS-7, many other circRNAs in eukaryotes have been suggested as potential miRNA sponges. For instance, circSRY, the testis-specific circRNA derived from sex-determining region Y (SRY), contained 16 MREs of miR-138 and acted as miR-138 sponge in mouse testis (12, 17). Also, circHIPK2 could work as a sponge for miR124-2HG to regulate the activation of astrocytes *via* the synergistic effect of autophagy and endoplasmic reticulum stress (45). In addition, circHIPK3, circCCDC66 and circPVT1 have been reported to have tumor-suppressive or oncogenic functions by serving as miRNA sponges (46–48). However, many circRNAs in mammals are expressed at relatively low levels and harbor few binding sites for miRNA (7, 49), suggesting them highly unlikely to act as ceRNAs or by working as miRNA sponges. Therefore, whether the miRNA sponge is a universal biological function of circRNAs needs to be further confirmed by more investigation.

### CircRNAs Interact With Proteins

In addition to miRNAs, circRNAs can also interact with proteins to form specific circRNA–protein complexes which affect the activity of related proteins (**Figure 2B**). The first example of circRNA acting as a protein sponge was a RNA transcript encoding the splicing factor protein–muscleblind (MBL) (34). The circular Mbl (circMbl) derived from its own gene contains multiple binding sites for MBL. This circRNA was found to regulate the linear splicing of the gene by functioning as a decoy for MBL. When MBL protein levels are high, MBL binds to the pre-mRNA and causes it to splice into circMbl. Subsequently, circMbl could sponge out the excess MBL by binding to it (34). Other circRNAs, such as circANRIL and circPABPN1, have also been reported to function as protein sponge by interacting with target proteins (50, 51). CircANRIL can bind to Pescadillo homologue 1 (PES1), a vital 60S pre-ribosomal assembly factor, and then dampens ribosome biogenesis and exonuclease-mediated pre-rRNA processing in human macrophages and vascular smooth muscle cells (50). CircPABPN1 represses the translation of nuclear poly (A) binding protein 1 mRNA in human cervical cancer (HeLa) cells through binding to the RNA binding protein Hu-antigen R (HUR) (51).

In some cases, circRNAs may function as protein scaffolds or alter modes of action of the proteins. For instance, circ-Foxo3 and circAmotl1 can function as scaffolds of protein to potentiate the colocalization of enzymes and their substrates (52, 53). In mouse cancer cells, circ-Foxo3 suppresses cell cycle progression by interacting with cyclin-dependent kinase 2 (CDK2) and cyclin-dependent kinase inhibitor 1 (p21) to form a circFoxo3-p21-CDK2 ternary complex, thereby arresting the functions of p21 and CDK2 in cell cycle regulation (52). In addition, circ-Foxo3 also has high binding affinity to senescence-related proteins ID-1 and E2F1 as well as anti-stress proteins FAK and HIF1a, and accelerates the location of these proteins in the cytoplasm and accordingly leads to increased cellular senescence (54). In a mouse model, circAmotl1 simultaneously binds to phosphoinositide- dependent kinase 1 (PDK1) and protein kinase AKT1 *via* its spatial structure, leading to PDK1-dependent AKT1 phosphorylation and nuclear translocation, thus enhancing the cardio-protective role of pAKT1 (53).

Although a growing number of circRNAs have been shown to interact with proteins, the exact mechanisms underlying these interactions remain obscure (55). Additionally, one general question is to what extent lowly expressed circRNAs can confer measurable regulation on their binding proteins.

### CircRNAs Can be Translatable

CircRNAs were initially believed to be non-coding RNA due to the absence of 5’ cap and 3’ ploy A tail that were normally required for linear mRNA translation. Given that most circRNAs were produced from protein-coding exons and mainly located in the cytoplasm. An intriguing question raises whether they are translatable. Recently, increasing evidence has shown that some circRNAs can be translatable molecules if they harbor an internal ribosome entry site (IRES) (56–58) (**Figure 2C**). In some cases, the presence of N6-methyladenosine (m6A) modification in the circRNAs can help to recruit the ribosome and promote the translation of circRNAs in cap-independent manner, but the underlying mechanisms remain largely unknown (59–61) (**Figure 2C**).

As early as the 1990s, Chen and Sarnow have proposed that synthetic engineered circRNAs may recruit ribosomal subunits and initiate peptide translation *in vitro* (62). However, this study did not support that circRNAs could also be translatable *in vivo*. In 2015, Abe and colleagues provided strong evidence that endogenous circRNAs can act as a template for translation in an IRES-dependent manner (56). Thereafter, several other groups have utilized mass spectrometry, ribosome profiling, and/or expression plasmids to demonstrate that a subset of circRNAs may indeed produce proteins (61, 63, 64). Nevertheless, to date only a few endogenous circRNAs, such as Mbl, circ-ZNF609, circFBXW7, cir-SHPRH, circPINTexon2, and circ-AKT3, have been reported to serve as protein templates (39, 63–67). Although thousands of circRNAs were predicted to have putative open reading frame (ORF) and IRES elements, not all of these circRNAs can perform translatingfunctions (68). The translational ability and mechanism of circRNAs are not fully understood and need to be further investigated. Furthermore, the functions of peptides/proteins encoded by circRNAs are still unknown. Since most circRNA-derived peptides are usually short peptides which lack important functional domains, they may function as decoys or dominant-negative protein variants (63). For example, several peptides, including FBXW-185aa, SHPRH-146aa, and PINT87aa, were reported to serve as tumor suppressors in glioblastoma (39, 65, 66). Although translation seems not to be a universal function for circRNAs, it is important to identify the physiological and pathological roles for the proteins translated by circRNAs.

### CircRNAs Regulate Transcription and Alternative Splicing

The CiRNAs and EIciRNAs, which consist of either introns or both intron and exon, are dominantly restricted in the nucleus. These circRNAs can regulate transcription through interaction with U1 small nuclear ribonucleoprotein (U1 snRNA) and the RNA polymerase II (Pol II) (31, 32) (**Figure 2D**). For instance, ci-ankrd52 can accumulate to its transcription sites and positively regulate the transcriptional activity of parental coding gene through association with elongation Pol II machinery (31). In addition, two ElcircRNAs, circEIF3J, and circPAIP2, were identified to interact with host U1 snRNP to form circRNA-U1 snRNP complexes. The complexes further interact with the Pol II to increase gene expression in the promoter region of parental genes (32).

CircRNA biosynthesis naturally affects host gene expression. From this point of view, two studies have shown that the circRNAs processing strongly competes with linear mRNA splicing (28, 34) (**Figure 2E**). Ashwal-Fluss et al. observed that circMbl derived from the second exon of Mbl can compete with conventional pre-mRNA to keep the balance between linear alternative splicing and circRNAs production (34). Another study has demonstrated that depletion or inhibition of canonical pre-mRNA processing events resulted in increased levels of circRNAs, suggesting that the negative association between circRNA and their linear mRNAs (28). Additionally, a nuclear-retained circSEP3 derived from SEPALLATA3 (SEP3) gene was reported to regulate its cognate mRNA splicing by forming an R-loop or RNA : DNA hybrid (69). Taken together, some nuclear retained circRNAs can participate in gene regulation in both transcription and splicing mechanisms. However, how these circRNAs are accumulated or localized in the nucleus and how exactly they modulate gene expression remains elusive.

## CircRNAs in Innate and Adaptive Immune Response

The immune system is classically divided into two categories, i.e. innate and adaptive immunity. Innate immune response is the first line of host defense against pathogens, such as antibacterial and antiviral immune responses. Adaptive immune response is the second line of defense against specific antigens. It creates immunological memories to the encountered antigens and initiates a highly efficient response to specific antigens in the future. Notably, recent studies have suggested that circRNAs are involved in the regulation of both innate and adaptive immune responses.

### CircRNAs in Innate Immunity

Innate immune response is mainly mediated by phagocytic cells and other cell types, such as macrophages, neutrophils, dendritic cells (DCs), and natural killer cells (NKs). In which, macrophages are one of the most important cell types in innate immune response. Through recognition of evolutionarily conserved structures on pathogens, macrophages become activated, and can engulf and destroy the invaders. Furthermore, macrophages can serve as antigen-presenting cells (APCs) to process and present foreign antigens during the adaptive immune response. According to the activation states and functions, macrophages can be divided into two subsets, i.e. classically activated macrophage (M1) and alternatively activated macrophage (M2). By performing a circRNA microarray analysis, Zhang et al. showed a total of 189 circRNAs were differentially expressed between M1 and M2 macrophages, indicating a role of circRNAs in macrophage polarization (70). Ng et al. reported that circ-RasGEF1B may play a regulatory role in macrophage activation by lipopolysaccharide (LPS) and protection against microbial infections (71). By comparing expression patterns of circRNAs in non-treated and RANKL/CSF1-treated bone marrow macrophage (BMM) cells, Chen et al. found that circRNA_28313 was significantly upregulated in RANK + CSF1-induced osteoclast differentiation by forming a ceRNA network of circRNA_28313/miR-195a/CSF1 (72). Circ-ASAP1 was reported to facilitate tumor-related macrophage infiltration by regulating miR-326/miR-532-5p-CSF-1 signaling pathway and may serve as a prognostic predict marker for hepatocellular carcinoma (73). By microarray analysis of circRNA expression profile in neutrophils derived from patients with asymptomatic moyamoya disease (MMD) and healthy individuals, 123 differentially expressed circRNAs were identified between the two groups. These differentially expressed circRNAs were mainly involved in immune responses, angiogenesis and metabolism in MMD (74). CircUHRF1 expression was elevated in human HCC tissues compared to adjacent non-tumor tissues. CircARSP91 may increase the susceptibility of HCC cells to NK cell cytotoxicity by upregulating UL16 binding protein 1 expression in HCC cells (75). More recently, Zhang et al. reported that tumor cell-derived exosomal circUHRF1 can induce NK cell exhaustion by inhibiting NK cell-derived IFN-γ and TNF-α secretion and may induce resistance to anti-PD1 therapy in human HCC (76). Expression of circRNA_001937 was significantly increased in PBMCs from patients with active tuberculosis and was positively correlated with disease severity (77). CircRNA-chr19 may function as a ceRNA by targeting miR-30b-3p/claudin-18 (CLDN18) axis in Ebola virus infection (78). Overexpression of a dsRNA-forming circRNA inhibited PKR activation and increased viral replication in encephalomyocarditis virus (EMCV) infection (79).

### CircRNAs in Adaptive Immunity

Adaptive immunity is characterized by T cells specific for antigens derived from the invading pathogens, and by B cells producing antibodies that bind to these antigens. T cells and B cells, once primed, initiate a highly efficient response to the pathogens and develop immunologic memories to the encountered antigens. The adaptive immune response is meant to defense foreign pathogens but sometimes it can mistakenly turn against our own body, leading to the autoimmune diseases.

Wang et al. reported that circRNA_100783 was involved in the regulation of CD8^+^ T cell ageing process by targeting a large number of miRNA-mRNA networks (80). Exosome-derived circRNA_002178 could be transferred into CD8^+^ T cells and promoted PD1 expression by sponging miR-28-5p, leading to CD8^+^ T cell exhaustion in lung adenocarcinoma (81). Huang et al. observed that circ_0005519 increased IL-13 and IL-6 expression *via* negatively regulating let-7a-5p in CD4^+^ T cells from patients with asthma (82). The same group also reported an increased expression of circ_0002594 in CD4^+^ T cells and might be a potential diagnostic marker for Th2-mediated allergic asthma (83). Gaffo et al. found that circRNAs displayed a cell type-specific expression pattern in healthy individuals, such as circRNAs specific for B-cells (circPAX5, circAFF3, circIL4R, and circSETBP1) and T-ceslls (circIKZF1, circTNIK, circTXK, and circFBXW7). The B cell-specific circRNAs circPAX5 and circAFF3 were highly overexpressed in pediatric B-precursor acute lymphoblastic leukemia (84). CircANKRD36 was significantly upregulated in peripheral blood and was correlated with chronic inflammation from patients with type 2 diabetes mellitus (T2DM) (85). Circ_402458 was significantly elevated in patients with primary biliary cholangitis (PBC). Circ_402458 may function as sponge for miR-522 and miR-943 and involved in the regulation of PBC (86).

## CircRNAs in SLE

**Figure 3:**
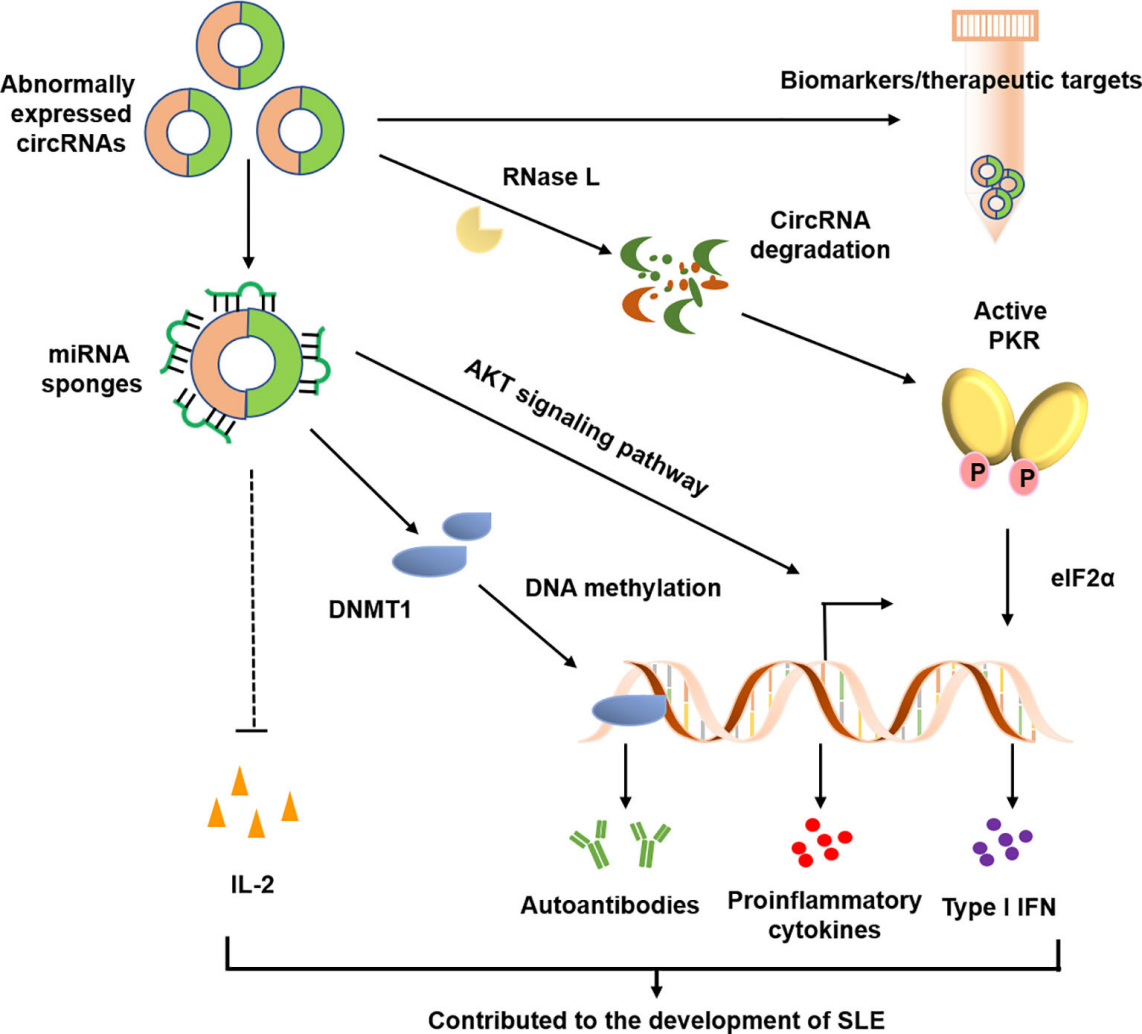
Possible roles of circRNAs in SLE. CircRANs may be involved in the pathogenesis of SLE by regulating various biological processes, such as DNA methylation, activation of AKT signaling pathway, and aberrant PKR activation. Furthermore, circRNAs may serve as biomarkers for the diagnosis and severity of SLE.

In the past three years, increasing evidence has linked circRNAs to SLE, with main focus on identification and characterization of circRNAs in blood samples, renal biopsies and skin tissues. These data suggest that circRNAs may be involved in the pathogenesis of SLE and could be potential diagnostic markers or therapeutic targets for SLE (**Figure 3** and **Table 1**).

**Table 1:** Summary of circRNAs implicated in SLE.

CircRNAs	Source/Detection Methods	CircRNA_types	Tissues	Dysregulation	Potential Functions/Applications	Refs
hsa_circRNA_400011 hsa_circRNA_102584 hsa_circRNA_101471 hsa_circRNA_100226	CircRNA microarray	Intronic Exonic Exonic Exonic	Plasma	Up Up Up Down	Possibly involved in the development of SLE by acting as miRNA sponges, may serve as potential biomarkers	(87)
hsa_circRNA_002453	CircRNA microarray	Not mentioned	Plasma	Up	Correlated with 24-hour proteinuria and renal SLEDAI score	(88)
hsa_circRNA_407176 hsa_circRNA_001308	CircRNA microarray	Not mentioned	Plasma	Down Down	Served as potential diagnostic biomarkers for SLE	(89)
hsa_circ_0057762 hsa_circ_0003090	CircRNA microarray	Not mentioned	Whole blood	Up Up	Served as potential diagnostic biomarkers for SLE	(90)
hsa_circ_0049224 hsa_circ_0049220	Selected from other studies	Not mentioned	PBMCs	Down Down	Negatively associated with SLEDAI and the degree of SLE severity	(91)
circIBTK	RNA-seq	Exonic	PBMCs	Down	Inhibited DNA demethylation and activation of AKT signaling pathway by binding to miR-29b, may serve as biomarkers and therapeutic targets for SLE	(92)
circPTPN22	RNA-seq	Exonic	PBMCs	Down	Acted as a biomarker for the diagnosis and severity of SLE	(93)
hsa_circ_0044235 hsa_circ_0068367	CircRNA microarray	Not mentioned	PBMCs	Up Up	Served as potential biomarkers for SLE diagnosis	(94)
hsa_circ_0000479	RNA-seq	Exonic	PBMCs	Up	Served as a novel diagnostic biomarker for SLE	(95)
hsa_circ_0000479	Selected from other studies	Exonic	PBMCs	Up	Served as a potential diagnostic biomarker for SLE	(96)
hsa_circ_0045272	CircRNA microarray	Not mentioned	T cells	Down	Upregulated the early apoptosis of Jurkat cells and promoted the production of IL-2 in activated Jurkat cells	(97)
hsa_circ_0012919	CircRNA microarray	Not mentioned	CD4+ T cells	Up	Inhibited DNA demethylation of CD11a and CD70 in CD4+ T cells, regulated the expression of RANTES and KLF13 *via* miR-125a-3p	(98)
circHLA-C	RNA-seq	Exonic	kidney	Up	Involved in the pathogenesis of lupus nephritis by sponging miR-150	(99)

### Peripheral Blood circRNAs in SLE

To date, the majority studies of circRNA in SLE were performed in blood samples, including plasma, whole blood, peripheral blood mononuclear cells (PBMCs), and T cells.

#### Plasma Derived circRNAs in SLE

As the high structural stability, the circRNAs are proposed to be promising biomarkers for diagnosis of diseases. By using circRNA microarray, Li et al. analyzed the circRNA expression profiling in plasma samples from patients with SLE and identified a number of circRNAs which were differentially expressed between SLE patients and healthy controls (87). By qPCR validation, four significantly dysregulated circRNAs, i.e., hsa_circ_400011, hsa_circ_102584, hsa_circ_101471, and hsa_circ_100226, were validated and predicted to be potential biomarkers for SLE diagnosis (87). Subsequently, Ouyang et al. reported that circRNA_002453 was markedly increased in plasma from patients with lupus nephritis (LN) compared with non-LN SLE patients, RA patients and healthy individuals (88). Although the expression level of circRNA_002453 had no significant association with C3, C4, and SLEDAI score, it was positively correlated with 24-hour proteinuria and renal SLEDAI score, which suggesting that this circRNA might severe as a novel biomarker for diagnosis of LN. Another research group has shown that hsa_circRNA_407176 and hsa_circRNA_001308 were underexpressed in both plasma and PBMCs of SLE patients (89). Furthermore, by the receiver operating characteristic (ROC) curve analysis, these two circRNAs could distinguish SLE patients from healthy controls, indicating their potential diagnostic value for SLE.

#### Whole Blood or PBMC Derived circRNAs in SLE

In a recent study, Li et al. analyzed the circRNA expression profile in whole blood from children with SLE (90). A total of 348 differentially and markedly expressed circRNAs were discovered, with 184 up-regulated and 164 down-regulated. They demonstrated that the levels of the hsa_circ_0057762 and hsa_circ_0003090 could distinguish the SLE patients from healthy individuals by ROC curve analysis. In the study, these authors constructed a comprehensive circRNA-miRNA-mRNA network, providing new insights into the molecular mechanisms of SLE pathogenesis in children (90). Zhang et al. initially focused on 2 circRNAs (hsa_circ_0049224 and hsa_circ_0049220), which have been demonstrated to be correlated with DNA methylation and the pathological processes of rheumatoid arthritis (RA) (100). Subsequently, they found that expression levels of hsa_circ_0049224 and hsa_circ_0049220 were significantly decreased in PBMCs of SLE patients and were negatively correlated with SLE disease activity index (SLEDAI) score (91). Moreover, the expression levels of these two circRNAs were positively correlated with the expression of DNMT1, a key factor of DNA methylation, which suggests their possible roles in the pathogenesis of SLE. In addition, the same group also performed circRNA sequencing to screen the circRNA expression profiles in PBMCs of patients with SLE (92). In this study, they observed a downregulation of circIBTK and upregulation of miR-29b in SLE patients, both were associated with SLEDAI score, titers of anti-dsDNA antibodies and levels of complement C3. Importantly, circIBTK could regulate DNA demethylation and the AKT signaling pathway by binding to miR-29b in SLE (92). Of note, numerous studies have demonstrated that aberrant DNA methylation and abnormal activation of AKT signaling pathway were involved in the pathogenesis of SLE (101–103). Furthermore, by RNA sequencing, Miao et al. identified 128 circRNAs that were aberrantly expressed in the PBMCs of SLE patients and discovered a novel circRNA, circPTPN22, might act as a potential biomarker for the diagnosis and disease severity of SLE (93). The decreased expression of circPTPN22 was negatively correlated with the SLEDAI scores in patients with SLE. The patients who received long-term corticosteroid therapy had significant increased expression levels circPTPN22. By using circRNAs microarray, Luo et al. also identified a number of differentially expressed circRNAs in PBMCs from patients with SLE (94). This study observed that the expression levels of hsa_circ_0044235 and hsa_circ_0068367 were markedly decreased in patients with SLE and these two circRNAs could be novel biomarkers for SLE diagnosis. The same group also reported that hsa_circ_0044235 was significantly downregulated in peripheral blood from RA patients and may serve as a potential biomarker for diagnosis of RA (104). In another study, Guo and colleagues reported that the hsa_circ_0000479 in PBMCs has a great potential as a diagnostic biomarker for SLE (95). However, the results from different circRNA profiling studies were inconsistent. Based on previous studies, Luo et al. selected 11 circRNAs which were upregulated in patients with SLE and validated the expression patterns and clinical significance of these circRNAs (96). Interestingly, the study replicated the elevated expression of hsa_circ_0000479 in PBMCs from SLE patients and its significant value in distinguishing SLE from other autoimmune diseases (96). A recent study has reported that a number of circRNAs were downregulated in PBMCs from patients with SLE. Meanwhile, the author also observed a spontaneous RNase L activation in PBMCs derived from SLE patients (79). The RNase L activation led to circRNA degradation and this RNase L-mediated circRNA degradation was required for protein kinase (PKR) activation. The aberrant PKR activation further augmented type I IFN signature through eIF2α phosphorylation and therefore involved in the pathogenesis of SLE (79).

#### T-Cell Derived circRNAs in SLE

T cells play a key role in lupus pathogenesis, especially the CD4 T helper (Th) cells (105). Li et al. recently reported 127 differentially expressed circRNAs in T cells from SLE patients, and further validated a downregulated circRNA, hsa_circ_0045272 (97). Further functional data indicated that knockdown of hsa_circ_0045272 resulted in an enhanced early apoptosis and increased IL-2 production in activated Jurkat cells (97). In another report, 12 up-regulated and two down-regulated circRNAs were discovered in CD4^+^ T cells of patients with SLE. Of which, three aberrantly up-regulated circRNAs, including hsa_circ_0012919, hsa_circ_0006239 and hsa_circ_0002227, were validated by qPCR (98). Of note, the hsa_circ_0012919 could be considered as a diagnostic potential for SLE. Downregulation of hsa_circ_0012919 led to an enhanced expression of DNA methyltransferase 1 and decreased expression of CD11a and CD70 on CD4^+^ T cells from both inactive and active SLE patients (98). Notably, the overexpression of CD11a and CD70 in CD4^+^ T cells in SLE may promote the production of autoantibodies (106). More recently, Zhang and colleagues analyzed 29 differentially expressed circRNAs obtained from the GSE84655 of GEO database (107). By statistical analysis and bioinformatic interpretation, the author predicted the circRNA-miRNA-mRNA regulatory networks for these circRNAs. These regulatory networks might be involved in the pathological process of SLE (107).

### Renal circRNAs in SLE

Lupus nephritis (LN) is one of the most serious complications of SLE. Patients with lupus nephritis are at risk for kidney failure and premature death. Through RNA deep sequencing, a total of 171 circRNAs with 2-fold differential expression, including 142 upregulated and 29 downregulated circRNAs, were discovered in renal biopsies from LN patients compared with normal kidney specimens (99). The correlations between seven validated circRNAs and clinical features were analyzed, and the circHLA-C was found to be positively associated with proteinuria, serum creatinine, renal activity index and crescentic glomeruli. Bioinformatic analysis predicted that circHLA-C plays important roles in the pathogenesis of lupus nephritis by serving as sponges for miR-150 and regulating its expression. The renal-derived miR-150 showed a tendency of negative correlation with circHLA-C in LN patients (99).

### Cutaneous circRNAs in SLE

Skin involvement is a common clinical manifestation in SLE patients. Of which, the discoid lupus erythematosus (DLE) is the most common form (108). By using RNA sequencing, Xuan and colleagues recently identified a number of differently expressed lncRNAs and circRNAs in the lesional lower lips from patients with DLE (109). In this study, a total of 161 circRNAs, including 57 up-regulated and 104 down-regulated circRNAs, showed remarkable differential expression in lesional skin compared with matched adjacent tissues. The principal roles of these markedly deregulated circRNAs were predicted using bioinformatics methods. These results suggested that certain circRNAs may have potential value in diagnosis and therapy of DLE.

## Conclusions and Perspectives

Recent advances in the circRNA research field have not only revealed a previously unexpected structure of eukaryotic transcriptomes but have also uncovered multiple aspects of circRNA biological function. CircRNAs generated by the non-canonical backsplicing can play important roles in biology and pathobiology by regulating gene expression at multiple molecular levels and interacting with proteins. These findings have been surprising and exciting, but there are still unrevealed answers concerning the biogenesis of these molecules. First, the complete set of elements that control the expression and localization of circRNAs remains unknown. For example, are there any factors that are indispensable for backsplicing but not for canonical splicing reactions? Second, the mechanisms involved in metabolic processing or degradation of circRNAs, particular in normal or unstressed cells, remain unclear. In addition, given the relatively low expression level of most circRNAs, how many circRNAs could truly act as miRNA sponges or competitors for protein binding?

The role of circRNAs in SLE has been in the spotlight in recent years. Several studies have shown that some aberrantly expressed circRNAs in SLE patients were implicated in DNA demethylation by acting as miRNA sponges (92, 98). However, the results were inconsistent from circRNA expression profiling studies of SLE PBMCs. These inconsistent results may be due to the disease heterogeneity and heterogeneous cell subsets in PBMCs such as monocytes, T cells and B cells. Therefore, it is important to stratify SLE patients by their clinical features and focus on certain cell subtypes. SLE is characterized by the production of a range of autoantibodies, indicating a critical role of B cells in the pathogenesis of SLE (2, 110). Therefore, it is worthy to functionally characterize the B cell-derived circRNAs involved in SLE pathogenesis. Additionally, a number of studies have shown that several circRNAs may serve as potential biomarkers for SLE. Nonetheless, the sample sizes in these studies were relatively small (95, 96). The results need to be further validated in larger sample sizes. More recently, an important study showed that overexpression of circPOLR2A, a double-stranded RNA (dsRNA)-containing circRNA, could remarkably attenuate the aberrant dsRNA-activated protein kinase activation cascade in PBMCs or T cells derived from SLE patients, suggesting that this type of circRNAs may serve as potential therapeutic modalities for SLE (79).

In conclusion, emerging evidence indicates that circRNAs are involved in the pathogenesis of SLE and may serve as novel biomarkers or therapeutics for SLE in the future. However, although the existing data support the potential value of circRNAs in diagnosis and treatment of SLE, the feasibility of circRNAs in clinical utility needs to be further studied.

## Author Contributions

LH wrote the manuscript. JG provided guidance, edited and revised the manuscript. ZY, CC, and YT helped in collecting the references and designed the figures. All authors contributed to the article and approved the submitted version.

## Funding

This work was supported in part by the National Natural Science Foundation of China (No. 31670915, No. 31870913, and No. 82071814 to JG, 81701614 to HL), and the University of Michigan Medical School (UMMS) and Peking University Health Science Center (PUHSC) Joint Institute (JI) Projects (No. BMU2020JI003 to JG).

## Conflict of Interest

The authors declare that the research was conducted in the absence of any commercial or financial relationships that could be construed as a potential conflict of interest.

## References

[1] Lisnevskaia L Murphy G Isenberg D. Systemic lupus erythematosus. Lancet (2014) 384:1878–88. 10.1016/s0140-6736(14)60128-8 24881804

[2] Tsokos GC. Systemic lupus erythematosus. N Engl J Med (2011) 365:2110–21. 10.1056/NEJMra1100359 22129255

[3] Xia X Tang X Wang S. Roles of CircRNAs in Autoimmune Diseases. Front Immunol (2019) 10:639. 10.3389/fimmu.2019.00639 31001261 PMC6454857

[4] Imamura K Akimitsu N. Long Non-Coding RNAs Involved in Immune Responses. Front Immunol (2014) 5:573:573. 10.3389/fimmu.2014.00573 25431574 PMC4230175

[5] Kristensen LS Andersen MS Stagsted LVW Ebbesen KK Hansen TB Kjems J. The biogenesis, biology and characterization of circular RNAs. Nat Rev Genet (2019) 20:675–91. 10.1038/s41576-019-0158-7 31395983

[6] Suzuki H Zuo Y Wang J Zhang MQ Malhotra A Mayeda A. Characterization of RNase R-digested cellular RNA source that consists of lariat and circular RNAs from pre-mRNA splicing. Nucleic Acids Res (2006) 34:e63. 10.1093/nar/gkl151 16682442 PMC1458517

[7] Enuka Y Lauriola M Feldman ME Sas-Chen A Ulitsky I Yarden Y. Circular RNAs are long-lived and display only minimal early alterations in response to a growth factor. Nucleic Acids Res (2016) 44:1370–83. 10.1093/nar/gkv1367 PMC4756822 26657629

[8] Sanger HL Klotz G Riesner D Gross HJ Kleinschmidt AK. Viroids are single-stranded covalently closed circular RNA molecules existing as highly base-paired rod-like structures. Proc Natl Acad Sci USA (1976) 73:3852–6. 10.1073/pnas.73.11.3852 PMC431239 1069269

[9] Hsu MT Coca-Prados M. Electron microscopic evidence for the circular form of RNA in the cytoplasm of eukaryotic cells. Nature (1979) 280:339–40. 10.1038/280339a0 460409

[10] Nigro JM Cho KR Fearon ER Kern SE Ruppert JM Oliner JD. Scrambled exons. Cell (1991) 64:607–13. 10.1016/0092-8674(91)90244-s 1991322

[11] Cocquerelle C Daubersies P Majérus MA Kerckaert JP Bailleul B. Splicing with inverted order of exons occurs proximal to large introns. EMBO J (1992) 11:1095–8. 10.1002/j.1460-2075.1992.tb05148.x PMC556550 1339341

[12] Capel B Swain A Nicolis S Hacker A Walter M Koopman P. Circular transcripts of the testis-determining gene Sry in adult mouse testis. Cell (1993) 73:1019–30. 10.1016/0092-8674(93)90279-y 7684656

[13] Cocquerelle C Mascrez B Hétuin D Bailleul B. Mis-splicing yields circular RNA molecules. FASEB J (1993) 7:155–60. 10.1096/fasebj.7.1.7678559 7678559

[14] Wang PL Bao Y Yee MC Barrett SP Hogan GJ Olsen MN. Circular RNA is expressed across the eukaryotic tree of life. PloS One (2014) 9:e90859. 10.1371/journal.pone.0090859 24609083 PMC3946582

[15] Jeck WR Sorrentino JA Wang K Slevin MK Burd CE Liu J. Circular RNAs are abundant, conserved, and associated with ALU repeats. RNA (2013) 19:141–57. 10.1261/rna.035667.112 PMC3543092 23249747

[16] Salzman J Chen RE Olsen MN Wang PL Brown PO. Cell-type specific features of circular RNA expression. PloS Genet (2013) 9:e1003777. 10.1371/journal.pgen.1003777 24039610 PMC3764148

[17] Hansen TB Jensen TI Clausen BH Bramsen JB Finsen B Damgaard CK. Natural RNA circles function as efficient microRNA sponges. Nature (2013) 495:384–8. 10.1038/nature11993 23446346

[18] Memczak S Jens M Elefsinioti A Torti F Krueger J Rybak A. Circular RNAs are a large class of animal RNAs with regulatory potency. Nature (2013) 495:333–8. 10.1038/nature11928 23446348

[19] Qu S Yang X Li X Wang J Gao Y Shang R. Circular RNA: A new star of noncoding RNAs. Cancer Lett (2015) 365:141–8. 10.1016/j.canlet.2015.06.003 26052092

[20] Nicolet BP Engels S Aglialoro F van den Akker E von Lindern M Wolkers MC. Circular RNA expression in human hematopoietic cells is widespread and cell-type specific. Nucleic Acids Res (2018) 46:8168–80. 10.1093/nar/gky721 PMC6144802 30124921

[21] Maass PG Glažar P Memczak S Dittmar G Hollfinger I Schreyer L. A map of human circular RNAs in clinically relevant tissues. J Mol Med (Berl) (2017) 95:1179–89. 10.1007/s00109-017-1582-9 PMC5660143 28842720

[22] Xia S Feng J Lei L Hu J Xia L Wang J. Comprehensive characterization of tissue-specific circular RNAs in the human and mouse genomes. Brief Bioinform (2017) 18:984–92. 10.1093/bib/bbw081 27543790

[23] Hanan M Soreq H Kadener S. CircRNAs in the brain. RNA Biol (2017) 14:1028–34. 10.1080/15476286.2016.1255398 PMC5680707 27892769

[24] Aufiero S Reckman YJ Pinto YM Creemers EE. Circular RNAs open a new chapter in cardiovascular biology. Nat Rev Cardiol (2019) 16:503–14. 10.1038/s41569-019-0185-2 30952956

[25] Vo JN Cieslik M Zhang Y Shukla S Xiao L Zhang Y. The Landscape of Circular RNA in Cancer. Cell (2019) 176:869–81.e13. 10.1016/j.cell.2018.12.021 30735636 PMC6601354

[26] Chen X Yang T Wang W Xi W Zhang T Li Q. Circular RNAs in immune responses and immune diseases. Theranostics (2019) 9:588–607. 10.7150/thno.29678 30809295 PMC6376182

[27] Salzman J Gawad C Wang PL Lacayo N Brown PO. Circular RNAs are the predominant transcript isoform from hundreds of human genes in diverse cell types. PloS One (2012) 7:e30733. 10.1371/journal.pone.0030733 22319583 PMC3270023

[28] Liang D Tatomer DC Luo Z Wu H Yang L Chen LL. The Output of Protein-Coding Genes Shifts to Circular RNAs When the Pre-mRNA Processing Machinery Is Limiting. Mol Cell (2017) 68:940–54.e3. 10.1016/j.molcel.2017.10.034 29174924 PMC5728686

[29] Jeck WR Sharpless NE. Detecting and characterizing circular RNAs. Nat Biotechnol (2014) 32:453–61. 10.1038/nbt.2890 PMC4121655 24811520

[30] Lasda E Parker R. Circular RNAs: diversity of form and function. RNA (2014) 20:1829–42. 10.1261/rna.047126.114 PMC4238349 25404635

[31] Zhang Y Zhang XO Chen T Xiang JF Yin QF Xing YH. Circular intronic long noncoding RNAs. Mol Cell (2013) 51:792–806. 10.1016/j.molcel.2013.08.017 24035497

[32] Li Z Huang C Bao C Chen L Lin M Wang X. Exon-intron circular RNAs regulate transcription in the nucleus. Nat Struct Mol Biol (2015) 22:256–64. 10.1038/nsmb.2959 25664725

[33] Conn SJ Pillman KA Toubia J Conn VM Salmanidis M Phillips CA. The RNA binding protein quaking regulates formation of circRNAs. Cell (2015) 160:1125–34. 10.1016/j.cell.2015.02.014 25768908

[34] Ashwal-Fluss R Meyer M Pamudurti NR Ivanov A Bartok O Hanan M. circRNA biogenesis competes with pre-mRNA splicing. Mol Cell (2014) 56:55–66. 10.1016/j.molcel.2014.08.019 25242144

[35] Rybak-Wolf A Stottmeister C Glazar P Jens M Pino N Giusti S. Circular RNAs in the Mammalian Brain Are Highly Abundant, Conserved, and Dynamically Expressed. Mol Cell (2015) 58:870–85. 10.1016/j.molcel.2015.03.027 25921068

[36] Errichelli L Dini Modigliani S Laneve P Colantoni A Legnini I Capauto D. FUS affects circular RNA expression in murine embryonic stem cell-derived motor neurons. Nat Commun (2017) 8:14741. 10.1038/ncomms14741 28358055 PMC5379105

[37] Zaiou M. Circular RNAs as Potential Biomarkers and Therapeutic Targets for Metabolic Diseases. Adv Exp Med Biol (2019) 1134:177–91. 10.1007/978-3-030-12668-1_10 30919338

[38] Lu Z Filonov GS Noto JJ Schmidt CA Hatkevich TL Wen Y. Metazoan tRNA introns generate stable circular RNAs in vivo. Rna (2015) 21:1554–65. 10.1261/rna.052944.115 PMC4536317 26194134

[39] Zhang M Zhao K Xu X Yang Y Yan S Wei P. A peptide encoded by circular form of LINC-PINT suppresses oncogenic transcriptional elongation in glioblastoma. Nat Commun (2018) 9:4475. 10.1038/s41467-018-06862-2 30367041 PMC6203777

[40] Bartel DP. MicroRNAs: genomics, biogenesis, mechanism, and function. Cell (2004) 116:281–97. 10.1016/s0092-8674(04)00045-5 14744438

[41] Salmena L Poliseno L Tay Y Kats L Pandolfi PP. A ceRNA hypothesis: the Rosetta Stone of a hidden RNA language? Cell (2011) 146:353–8. 10.1016/j.cell.2011.07.014 PMC3235919 21802130

[42] Poliseno L Salmena L Zhang J Carver B Haveman WJ Pandolfi PP. A coding-independent function of gene and pseudogene mRNAs regulates tumour biology. Nature (2010) 465:1033–8. 10.1038/nature09144 PMC3206313 20577206

[43] Cesana M Cacchiarelli D Legnini I Santini T Sthandier O Chinappi M. A long noncoding RNA controls muscle differentiation by functioning as a competing endogenous RNA. Cell (2011) 147:358–69. 10.1016/j.cell.2011.09.028 PMC3234495 22000014

[44] Li RC Ke S Meng FK Lu J Zou XJ He ZG. CiRS-7 promotes growth and metastasis of esophageal squamous cell carcinoma via regulation of miR-7/HOXB13. Cell Death Dis (2018) 9:838. 10.1038/s41419-018-0852-y 30082829 PMC6079012

[45] Huang R Zhang Y Han B Bai Y Zhou R Gan G. Circular RNA HIPK2 regulates astrocyte activation via cooperation of autophagy and ER stress by targeting MIR124-2HG. Autophagy (2017) 13:1722–41. 10.1080/15548627.2017.1356975 PMC5640207 28786753

[46] Zheng Q Bao C Guo W Li S Chen J Chen B. Circular RNA profiling reveals an abundant circHIPK3 that regulates cell growth by sponging multiple miRNAs. Nat Commun (2016) 7:11215. 10.1038/ncomms11215 27050392 PMC4823868

[47] Hsiao K-Y Lin Y-C Gupta SK Chang N Yen L Sun HS. Noncoding Effects of Circular RNA CCDC66 Promote Colon Cancer Growth and Metastasis. Cancer Res (2017) 77:2339–50. 10.1158/0008-5472.CAN-16-1883 PMC5910173 28249903

[48] Verduci L Ferraiuolo M Sacconi A Ganci F Vitale J Colombo T. The oncogenic role of circPVT1 in head and neck squamous cell carcinoma is mediated through the mutant p53/YAP/TEAD transcription-competent complex. Genome Biol (2017) 18:237. 10.1186/s13059-017-1368-y 29262850 PMC5738800

[49] Guo JU Agarwal V Guo H Bartel DP. Expanded identification and characterization of mammalian circular RNAs. Genome Biol (2014) 15:409. 10.1186/s13059-014-0409-z 25070500 PMC4165365

[50] Holdt LM Stahringer A Sass K Pichler G Kulak NA Wilfert W. Circular non-coding RNA ANRIL modulates ribosomal RNA maturation and atherosclerosis in humans. Nat Commun (2016) 7:12429. 10.1038/ncomms12429 27539542 PMC4992165

[51] Abdelmohsen K Panda AC Munk R Grammatikakis I Dudekula DB De S. Identification of HuR target circular RNAs uncovers suppression of PABPN1 translation by CircPABPN1. RNA Biol (2017) 14:361–9. 10.1080/15476286.2017.1279788 PMC5367248 28080204

[52] Du WW Yang W Liu E Yang Z Dhaliwal P Yang BB. Foxo3 circular RNA retards cell cycle progression via forming ternary complexes with p21 and CDK2. Nucleic Acids Res (2016) 44:2846–58. 10.1093/nar/gkw027 PMC4824104 26861625

[53] Du WW Yang W Chen Y Wu ZK Foster FS Yang Z. Foxo3 circular RNA promotes cardiac senescence by modulating multiple factors associated with stress and senescence responses. Eur Heart J (2017) 38:1402–12. 10.1093/eurheartj/ehw001 26873092

[54] Zeng Y Du WW Wu Y Yang Z Awan FM Li X. A Circular RNA Binds To and Activates AKT Phosphorylation and Nuclear Localization Reducing Apoptosis and Enhancing Cardiac Repair. Theranostics (2017) 7:3842–55. 10.7150/thno.19764 PMC5667408 29109781

[55] Huang A Zheng H Wu Z Chen M Huang Y. Circular RNA-protein interactions: functions, mechanisms, and identification. Theranostics (2020) 10:3503–17. 10.7150/thno.42174 PMC7069073 32206104

[56] Abe N Matsumoto K Nishihara M Nakano Y Shibata A Maruyama H. Rolling Circle Translation of Circular RNA in Living Human Cells. Sci Rep (2015) 5:16435. 10.1038/srep16435 26553571 PMC4639774

[57] Wang Y Wang Z. Efficient backsplicing produces translatable circular mRNAs. Rna (2015) 21:172–9. 10.1261/rna.048272.114 PMC4338345 25449546

[58] Wesselhoeft RA Kowalski PS Anderson DG. Engineering circular RNA for potent and stable translation in eukaryotic cells. Nat Commun (2018) 9:2629. 10.1038/s41467-018-05096-6 29980667 PMC6035260

[59] Meyer KD Patil DP Zhou J Zinoviev A Skabkin MA Elemento O. 5’ UTR m(6)A Promotes Cap-Independent Translation. Cell (2015) 163:999–1010. 10.1016/j.cell.2015.10.012 26593424 PMC4695625

[60] Zhou J Wan J Gao X Zhang X Jaffrey SR Qian SB. Dynamic m(6)A mRNA methylation directs translational control of heat shock response. Nature (2015) 526:591–4. 10.1038/nature15377 PMC4851248 26458103

[61] Yang Y Fan X Mao M Song X Wu P Zhang Y. Extensive translation of circular RNAs driven by N(6)-methyladenosine. Cell Res (2017) 27:626–41. 10.1038/cr.2017.31 PMC5520850 28281539

[62] Chen CY Sarnow P. Initiation of protein synthesis by the eukaryotic translational apparatus on circular RNAs. Science (1995) 268:415–7. 10.1126/science.7536344 7536344

[63] Legnini I Di Timoteo G Rossi F Morlando M Briganti F Sthandier O. Circ-ZNF609 Is a Circular RNA that Can Be Translated and Functions in Myogenesis. Mol Cell (2017) 66:22–37 e9. 10.1016/j.molcel.2017.02.017 28344082 PMC5387670

[64] Pamudurti NR Bartok O Jens M Ashwal-Fluss R Stottmeister C Ruhe L. Translation of CircRNAs. Mol Cell (2017) 66:9–21.e7. 10.1016/j.molcel.2017.02.021 28344080 PMC5387669

[65] Yang Y Gao X Zhang M Yan S Sun C Xiao F. Novel Role of FBXW7 Circular RNA in Repressing Glioma Tumorigenesis. J Natl Cancer Inst (2018) 110:304–15. 10.1093/jnci/djx166 PMC6019044 28903484

[66] Zhang M Huang N Yang X Luo J Yan S Xiao F. A novel protein encoded by the circular form of the SHPRH gene suppresses glioma tumorigenesis. Oncogene (2018) 37:1805–14. 10.1038/s41388-017-0019-9 29343848

[67] Xia X Li X Li F Wu X Zhang M Zhou H. A novel tumor suppressor protein encoded by circular AKT3 RNA inhibits glioblastoma tumorigenicity by competing with active phosphoinositide-dependent Kinase-1. Mol Cancer (2019) 18:131. 10.1186/s12943-019-1056-5 31470874 PMC6716823

[68] Chen X Han P Zhou T Guo X Song X Li Y. circRNADb: A comprehensive database for human circular RNAs with protein-coding annotations. Sci Rep (2016) 6:34985. 10.1038/srep34985 27725737 PMC5057092

[69] Conn VM Hugouvieux V Nayak A Conos SA Capovilla G Cildir G. A circRNA from SEPALLATA3 regulates splicing of its cognate mRNA through R-loop formation. Nat Plants (2017) 3:17053. 10.1038/nplants.2017.53 28418376

[70] Zhang Y Zhang Y Li X Zhang M Lv K. Microarray analysis of circular RNA expression patterns in polarized macrophages. Int J Mol Med (2017) 39:373–9. 10.3892/ijmm.2017.2852 PMC5358696 28075448

[71] Ng WL Marinov GK Chin YM Lim YY Ea CK. Transcriptomic analysis of the role of RasGEF1B circular RNA in the TLR4/LPS pathway. Sci Rep (2017) 7:12227. 10.1038/s41598-017-12550-w 28947785 PMC5612941

[72] Chen X Ouyang Z Shen Y Liu B Zhang Q Wan L. CircRNA_28313/miR-195a/CSF1 axis modulates osteoclast differentiation to affect OVX-induced bone absorption in mice. RNA Biol (2019) 16:1249–62. 10.1080/15476286.2019.1624470 PMC6693548 31204558

[73] Hu ZQ Zhou SL Li J Zhou ZJ Wang PC Xin HY. Circular RNA Sequencing Identifies CircASAP1 as a Key Regulator in Hepatocellular Carcinoma Metastasis. Hepatology (2020) 72:906–22. 10.1002/hep.31068 31838741

[74] Ma Q Li L Yu B Jiao L Han Z Zhao H. Circular RNA profiling of neutrophil transcriptome provides insights into asymptomatic Moyamoya disease. Brain Res (2019) 1719:104–12. 10.1016/j.brainres.2019.05.033 31132337

[75] Ma Y Zhang C Zhang B Yu H Yu Q. circRNA of AR-suppressed PABPC1 91 bp enhances the cytotoxicity of natural killer cells against hepatocellular carcinoma via upregulating UL16 binding protein 1. Oncol Lett (2019) 17:388–97. 10.3892/ol.2018.9606 PMC6313186 30655779

[76] Zhang PF Gao C Huang XY Lu JC Guo XJ Shi GM. Cancer cell-derived exosomal circUHRF1 induces natural killer cell exhaustion and may cause resistance to anti-PD1 therapy in hepatocellular carcinoma. Mol Cancer (2020) 19:110. 10.1186/s12943-020-01222-5 32593303 PMC7320583

[77] Huang ZK Yao FY Xu JQ Deng Z Su RG Peng YP. Microarray Expression Profile of Circular RNAs in Peripheral Blood Mononuclear Cells from Active Tuberculosis Patients. Cell Physiol Biochem (2018) 45:1230–40. 10.1159/000487454 29448254

[78] Wang ZY Guo ZD Li JM Zhao ZZ Fu YY Zhang CM. Genome-Wide Search for Competing Endogenous RNAs Responsible for the Effects Induced by Ebola Virus Replication and Transcription Using a trVLP System. Front Cell Infect Microbiol (2017) 7:479:479. 10.3389/fcimb.2017.00479 29209594 PMC5702449

[79] Liu CX Li X Nan F Jiang S Gao X Guo SK. Structure and Degradation of Circular RNAs Regulate PKR Activation in Innate Immunity. Cell (2019) 177:865–80.e21. 10.1016/j.cell.2019.03.046 31031002

[80] Wang YH Yu XH Luo SS Han H. Comprehensive circular RNA profiling reveals that circular RNA100783 is involved in chronic CD28-associated CD8(+)T cell ageing. Immun Ageing (2015) 12:17. 10.1186/s12979-015-0042-z 26451160 PMC4597608

[81] Wang J Zhao X Wang Y Ren F Sun D Yan Y. circRNA-002178 act as a ceRNA to promote PDL1/PD1 expression in lung adenocarcinoma. Cell Death Dis (2020) 11:32. 10.1038/s41419-020-2230-9 31949130 PMC6965119

[82] Huang Z Cao Y Zhou M Qi X Fu B Mou Y. Hsa_circ_0005519 increases IL-13/IL-6 by regulating hsa-let-7a-5p in CD4(+) T cells to affect asthma. Clin Exp Allergy (2019) 49:1116–27. 10.1111/cea.13445 31148290

[83] Huang Z Fu B Qi X Xu Y Mou Y Zhou M. Diagnostic and Therapeutic Value of Hsa_circ_0002594 for T Helper 2-Mediated Allergic Asthma. Int Arch Allergy Immunol (2020) 1–11. 10.1159/000511612 33326955

[84] Gaffo E Boldrin E Dal Molin A Bresolin S Bonizzato A Trentin L. Circular RNA differential expression in blood cell populations and exploration of circRNA deregulation in pediatric acute lymphoblastic leukemia. Sci Rep (2019) 9:14670. 10.1038/s41598-019-50864-z 31605010 PMC6789028

[85] Fang Y Wang X Li W Han J Jin J Su F. Screening of circular RNAs and validation of circANKRD36 associated with inflammation in patients with type 2 diabetes mellitus. Int J Mol Med (2018) 42:1865–74. 10.3892/ijmm.2018.3783 PMC6108858 30066828

[86] Zheng J Li Z Wang T Zhao Y Wang Y. Microarray Expression Profile of Circular RNAs in Plasma from Primary Biliary Cholangitis Patients. Cell Physiol Biochem (2017) 44:1271–81. 10.1159/000485487 29183005

[87] Li H Li K Lai W Li X Wang H Yang J. Comprehensive circular RNA profiles in plasma reveals that circular RNAs can be used as novel biomarkers for systemic lupus erythematosus. Clin Chim Acta (2018) 480:17–25. 10.1016/j.cca.2018.01.026 29360436

[88] Ouyang Q Huang Q Jiang Z Zhao J Shi GP Yang M. Using plasma circRNA_002453 as a novel biomarker in the diagnosis of lupus nephritis. Mol Immunol (2018) 101:531–8. 10.1016/j.molimm.2018.07.029 30172209

[89] Zhang MY Wang JB Zhu ZW Li LJ Liu RS Yang XK. Differentially expressed circular RNAs in systemic lupus erythematosus and their clinical significance. BioMed Pharmacother (2018) 107:1720–7. 10.1016/j.biopha.2018.08.161 30257390

[90] Li S Zhang J Tan X Deng J Li Y Piao Y. Microarray expression profile of circular RNAs and mRNAs in children with systemic lupus erythematosus. Clin Rheumatol (2019) 38:1339–50. 10.1007/s10067-018-4392-8 30628013

[91] Zhang C Huang J Chen Y Shi W. Low Expression and Clinical Value of hsa_circ_0049224 and has_circ_0049220 in Systemic Lupus Erythematous Patients. Med Sci Monit (2018) 24:1930–5. 10.12659/msm.906507 PMC5898388 29606700

[92] Wang X Zhang C Wu Z Chen Y Shi W. CircIBTK inhibits DNA demethylation and activation of AKT signaling pathway via miR-29b in peripheral blood mononuclear cells in systemic lupus erythematosus. Arthritis Res Ther (2018) 20:118. 10.1186/s13075-018-1618-8 29884225 PMC5993996

[93] Miao Q Zhong Z Jiang Z Lin Y Ni B Yang W. RNA-seq of circular RNAs identified circPTPN22 as a potential new activity indicator in systemic lupus erythematosus. Lupus (2019) 28:520–8. 10.1177/0961203319830493 30871426

[94] Luo Q Zhang L Li X Fu B Guo Y Huang Z. Identification of circular RNAs hsa_circ_0044235 and hsa_circ_0068367 as novel biomarkers for systemic lupus erythematosus. Int J Mol Med (2019) 44:1462–72. 10.3892/ijmm.2019.4302 PMC6713423 31432107

[95] Guo G Wang H Ye L Shi X Yan K Lin K. Hsa_circ_0000479 as a Novel Diagnostic Biomarker of Systemic Lupus Erythematosus. Front Immunol (2019) 10:2281:2281. 10.3389/fimmu.2019.02281 31608065 PMC6771011

[96] Luo Q Zhang L Fang L Fu B Guo Y Huang Z. Circular RNAs hsa_circ_0000479 in peripheral blood mononuclear cells as novel biomarkers for systemic lupus erythematosus. Autoimmunity (2020) 53:167–76. 10.1080/08916934.2020.1728529 32093518

[97] Li LJ Zhu ZW Zhao W Tao SS Li BZ Xu SZ. Circular RNA expression profile and potential function of hsa_circ_0045272 in systemic lupus erythematosus. Immunology (2018) 155:137–49. 10.1111/imm.12940 PMC6099170 29700819

[98] Zhang C Wang X Chen Y Wu Z Zhang C Shi W. The down-regulation of hsa_circ_0012919, the sponge for miR-125a-3p, contributes to DNA methylation of CD11a and CD70 in CD4(+) T cells of systemic lupus erythematous. Clin Sci (Lond) (2018) 132:2285–98. 10.1042/CS20180403 30237316

[99] Luan J Jiao C Kong W Fu J Qu W Chen Y. circHLA-C Plays an Important Role in Lupus Nephritis by Sponging miR-150. Mol Ther Nucleic Acids (2018) 10:245–53. 10.1016/j.omtn.2017.12.006 PMC5768151 29499937

[100] Song X Zhang L Zhao S Wang X Wang L Chen L. Peripheral blood circRNA expression profile analysis in patients with rheumatoid arthritis. Chin J Rheumatol (2016) 20:541–6.

[101] Wu H Zhao M Tan L Lu Q. The key culprit in the pathogenesis of systemic lupus erythematosus: Aberrant DNA methylation. Autoimmun Rev (2016) 15:684–9. 10.1016/j.autrev.2016.03.002 26970492

[102] Tang H Tan G Guo Q Pang R Zeng F. Abnormal activation of the Akt-GSK3beta signaling pathway in peripheral blood T cells from patients with systemic lupus erythematosus. Cell Cycle (2009) 8:2789–93. 10.4161/cc.8.17.9446 19652548

[103] Taher TE Parikh K Flores-Borja F Mletzko S Isenberg DA Peppelenbosch MP. Protein phosphorylation and kinome profiling reveal altered regulation of multiple signaling pathways in B lymphocytes from patients with systemic lupus erythematosus. Arthritis Rheumatol (2010) 62:2412–23. 10.1002/art.27505 20506108

[104] Luo Q Zhang L Li X Fu B Deng Z Qing C. Identification of circular RNAs hsa_circ_0044235 in peripheral blood as novel biomarkers for rheumatoid arthritis. Clin Exp Immunol (2018) 194:118–24. 10.1111/cei.13181 PMC6156811 30216431

[105] Crispín JC Kyttaris VC Terhorst C Tsokos GC. T cells as therapeutic targets in SLE. Nat Rev Rheumatol (2010) 6:317–25. 10.1038/nrrheum.2010.60 PMC2924434 20458333

[106] Zhao M Sun Y Gao F Wu X Tang J Yin H. Epigenetics and SLE: RFX1 downregulation causes CD11a and CD70 overexpression by altering epigenetic modifications in lupus CD4+ T cells. J Autoimmun (2010) 35:58–69. 10.1016/j.jaut.2010.02.002 20223637

[107] Zhang J Liu Y Shi G. The circRNA-miRNA-mRNA regulatory network in systemic lupus erythematosus. Clin Rheumatol (2020) 40:331–39. 10.1007/s10067-020-05212-2 32533339

[108] Okon LG Werth VP. Cutaneous lupus erythematosus: diagnosis and treatment. Best Pract Res Clin Rheumatol (2013) 27:391–404. 10.1016/j.berh.2013.07.008 24238695 PMC3927537

[109] Xuan J Xiong Y Shi L Aramini B Wang H. Do lncRNAs and circRNAs expression profiles influence discoid lupus erythematosus progression?-a comprehensive analysis. Ann Transl Med (2019) 7:728. 10.21037/atm.2019.12.10 32042744 PMC6990042

[110] Scharer CD Blalock EL Mi T Barwick BG Jenks SA Deguchi T. Epigenetic programming underpins B cell dysfunction in human SLE. Nat Immunol (2019) 20:1071–82. 10.1038/s41590-019-0419-9 PMC6642679 31263277

